# Comparison of Conventionally Performed and Intracardiac Echocardiography Guided Catheter Ablation of Atrioventricular Node in Patients with Permanent Atrial Fibrillation—A Retrospective Single-Center Study

**DOI:** 10.3390/jcm13154565

**Published:** 2024-08-05

**Authors:** Dorottya Debreceni, Maja Mandel, Kristof-Ferenc Janosi, Botond Bocz, Dalma Torma, Tamas Simor, Peter Kupo

**Affiliations:** Heart Institute, Medical School, University of Pecs, H-7624 Pecs, Hungaryhtqb2l@tr.pte.hu (B.B.); sjmlql@tr.pte.hu (D.T.); simor.tamas@pte.hu (T.S.)

**Keywords:** intracardiac echocardiography, atrioventricular node ablation, minimal fluoroscopy, atrial fibrillation

## Abstract

**Background:** Atrioventricular node (AVN) ablation is an effective treatment for atrial fibrillation (AF) with uncontrolled ventricular rates despite maximal pharmacological treatment. Intracardiac echocardiography (ICE) can help with visualizing structures, positioning catheters, and guiding the ablation procedure. We compared only fluoroscopy-guided and ICE-guided AVN ablation regarding patients with permanent AF. **Methods:** Sixty-two consecutive patients underwent AVN ablation were enrolled in our retrospective single-center study (ICE group: 28 patients, Standard group: 34 patients). Procedural data, acute and long-term success rate, and complications were analyzed. **Results:** ICE guidance for AVN ablation significantly reduced fluoroscopy time (0.30 [0.06; 0.85] min vs. 7.95 [3.23; 6.59] min, *p* < 0.01), first-to-last ablation time (4 [2; 16.3] min vs. 26.5 [2.3; 72.5] min, *p* = 0.02), and in-procedure time (40 [34; 55] min vs. 60 [45; 110], *p* = 0.02). There was no difference in either the total ablation time (199 [91; 436] s vs. 294 [110; 659] s, *p* = 0.22) or in total ablation energy (8272 [4004; 14,651] J vs. 6065 [2708; 16,406] J, *p* = 0.28). The acute success rate was similar (ICE: 100% vs. Standard: 94%, *p* = 0.49) between the groups. **Conclusions:** In our retrospective trial, ICE-guided AVN ablation reduced fluoroscopy time, procedure time, and first-to-last ablation time. There was no difference in ablation time, total ablation energy, acute and long-term success, and complication rate.

## 1. Introduction

Atrioventricular node ablation (AVNA) with permanent pacemaker implantation is a recommended choice of rate control therapy for patients with permanent atrial fibrillation (AF) when intensive pharmacological treatments prove ineffective [1]. Recent evidence indicates that employing pace and ablate strategies are superior to pharmacological therapy in decreasing hospitalization rates and reducing mortality among patients for whom a rhythm control strategy has proved ineffective [2]. AVNA has a low complication rate and high acute and long-term success rate; a prospective registry showed that only 3.5% of patients who underwent AVNA experienced a recurrence of AV conduction during the extended observational period [3].

In AVNA procedures, the positioning of the ablation catheter is guided by anatomical considerations, and intracardiac electrocardiograms are used to verify precision [4]. Conventionally, catheter manipulation is guided by fluoroscopy during invasive electrophysiology procedures, thereby exposing both patients and medical staff to a potentially dangerous amount of ionizing radiation, which is associated with potential long-term health risks [5,6].

Intracardiac echocardiography (ICE) provides real-time imaging of the heart’s anatomical structures, enabling invasive cardiologists to visualize catheter placement and monitor tissue changes; moreover, ICE detects major procedure-related complications (i.e., pericardial effusion). Furthermore, several recent studies have shown that usage of ICE in electrophysiology procedures significantly reduces procedural and fluoroscopy time; this leads to significantly reduced radiation exposure and a shorter duration for ablation compared to procedures relying solely on fluoroscopy [7,8].

The aim of our single-center, retrospective study was to estimate the effect of ICE guidance on procedural outcomes in patients with permanent AF who underwent AVNA.

## 2. Methods

### 2.1. Study Protocol

In this retrospective, single-center trial, we involved a cohort of 62 patients who underwent AVNA for permanent AF with an uncontrolled ventricular rate despite maximal dose pharmacological treatment at our university hospital between January 2015 and January 2024. The patients were categorized into two distinct groups based on the utilization of ICE during the procedure, specifically the ICE group and the Standard group, for subsequent comparative analysis. ICE usage depended on the operators’ discretion. Patients referred for a repeat (redo) procedure were excluded from the study, as were cases involving crossovers where ICE was not initially utilized at the onset of the procedure but was subsequently introduced later in the process due to the unsuccessful achievement of the procedural endpoint.

The study protocol strictly followed the principles outlined in the Declaration of Helsinki. Ethical approval was obtained in accordance with the institutional research ethics board, and all participants provided informed consent. Before the intervention, all patients underwent a comprehensive initial clinical evaluation, including a review of medical history, electrocardiography, routine blood tests, and echocardiography.

### 2.2. AV Node Ablation Procedures

Catheter ablation procedures were conducted with conscious sedation, utilizing midazolam +/− fentanyl, and patients maintained a fasting state while adhering to an uninterrupted anticoagulation therapy. The procedures were performed by three skilled electrophysiologists with limited experience in ICE-guided AVNA techniques but possessing significant expertise in fluoroscopy-guided AVNA ablations. Vascular ultrasound guidance for femoral vessel punctures in our center has been utilized since January 2021; consequently, femoral vein punctures for procedures performed between 2015 and 2021 were conducted without ultrasound guidance. Following local anesthesia and femoral venous access, an irrigated 4 mm tip ablation catheter (Alcath Black Flux G, Biotronik, Berlin, Germany) was introduced into the right atrium. The decision to use the 8F ICE catheter (AcuNaV™ 90 cm, Siemens Medical Solutions, Mountain View, CA, USA) was left to the operators’ consideration.

Twelve-lead electrocardiogram recordings and intracardiac electrograms were acquired and stored operating a digital recording system (CardioLab, GE Healthcare, Chicago, IL, USA).

**Figure 1:**
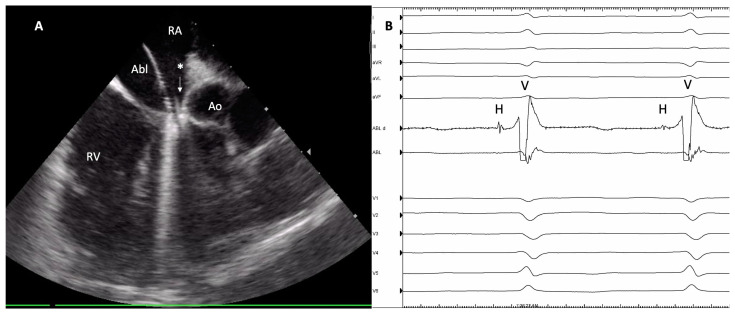
(**A**): Intracardiac ultrasound (ICE) visualization of the ablation catheter in the target area during atrioventricular node (AVN) ablation. * represents the previously implanted pacemaker lead. (**B**): Intracardiac electrocardiogram recorded by the ablation catheter (Abl) simultaneously with the twelve-lead surface electrocardiogram during ongoing atrial fibrillation. Abbreviations: Abl: ablation catheter, Ao: aortic annulus, H: local His potential, RA: right atrium, RV: right ventricle.

After catheter placement, the upper third of the Koch triangle region was mapped. RF ablation was performed when a His electrogram was recorded with the ablation catheter. The ablation procedure was performed under temperature-controlled conditions, aiming for a target temperature of 43 °C. Power delivery was constrained to 40–50 W, with continuous irrigation maintained at a rate of 15 mL/min. If the procedural endpoint was not achieved following numerous right-sided RF ablations, operators exercised discretion to proceed with transseptal puncture and a left-sided approach. In the ICE-guided procedures, the catheter was positioned in the inferior right atrium at the 4 o’clock orientation, allowing for lateral positioning or orientation in the direction of the septum as needed (Figure 1). This setup enabled the visualization of anatomical landmarks in the lower right atrium, including structures such as the septal right ventricle, aortic root, and tricuspid valve (Supplementary Video S1). In cases where ICE was utilized, an additional femoral vein puncture was performed at the beginning of the procedure.

The procedural endpoint was defined as complete AV block after a 20 min waiting period from the last ablation. Long-term success was defined as persistent complete AV block 30 days after the procedure. Major complications were identified as pericardial tamponade/effusion or vascular complications necessitating intervention or extended hospitalization, such as major hematomas, pseudoaneurysms, and arteriovenous fistulas. The procedure duration, quantified in minutes, spanned from the initial femoral vein puncture to the removal of the last catheter. The duration of ablation procedures, measured in minutes, was calculated from the commencement to the conclusion of the final RF application. Fluoroscopy time, also in minutes, and radiation dose were systematically documented by the fluoroscopy system. The ablation duration, recorded in seconds, was measured using the EP recording system.

### 2.3. Statistical Analysis

The attributes of data distribution were evaluated using the Shapiro–Wilk test. All statistical analyses were performed using a two-tailed methodology, with a significance threshold set at *p* < 0.05. Continuous variables were represented either as the mean ± standard deviation or the median with interquartile range, depending on the most suitable representation. In contrast, categorical variables were expressed in absolute numbers and percentages. Appropriate statistical tests, including the Chi-square test, t-test, and Mann–Whitney U test, were employed for comparative analyses. All statistical procedures were conducted using SPSS version 28 software (SPSS Inc., Chicago, IL, USA).

## 3. Results

A total of 62 participants were enrolled. Overall, 28 patients underwent ICE-guided AVNA, while in 34 cases, fluoroscopy was used exclusively. The baseline demographic characteristics, including sex distribution (male: 64% vs. 59%, *p* = 0.66) and age (72.3 ± 9 vs. 68.3 ± 8.8 years, *p* = 0.08), showed no significant differences between the two groups (Table 1).

ICE guidance was associated with a shorter procedure (40 [34; 55] min vs. 60 [45; 110], *p* = 0.02) and fluoroscopy time (0.30 [0.06; 0.85] min vs. 7.95 [3.23; 6.59] min, *p* < 0.01). Similarly, the time from the first to the last ablation (4 [2; 16.3] min vs. 26.5 [2.3; 72.5] min, *p* = 0.02), total ablation time (199 [91; 436] s vs. 294 [110; 659] s, *p* = 0.22), and total ablation energy (8272 [4004; 14,651] J vs. 6065 [2708; 16,406] J, *p* = 0.28) were reduced in the ICE group.

The acute success rate was similar between the groups (ICE: 100% vs. Standard: 94%, *p* = 0.49), while recurrent AV nodal conduction was reported in one patient in each group (*p* = 0.94). One complication occurred in the ICE group during the study period, which was a major hematoma related to the femoral puncture site and required vascular surgery (*p* = 0.94). Results are summarized in Table 2.

## 4. Discussion

In this retrospective single-center study, we observed that utilizing ICE in AVNA significantly decreased fluoroscopy time, overall procedure time, and the first-to-last ablation time, without compromising acute and long-term outcomes and complication rates.

According to the 2020 guidelines from the European Society of Cardiology for the diagnosis and management of atrial fibrillation, catheter ablation is deemed crucial in the treatment of permanent AF that remains unresponsive to intensive pharmacological frequency control [1]. The irreversible damage to the conduction system and results in pacemaker dependency, which can lead to heart failure (HF) when right ventricular (RV) pacing is used. Although it can be stated retrospectively that the majority of patients in both groups received a VVI pacemaker before AVN ablation, our study did not examine the types of pacemakers implanted and their impact on subsequent quality of life. In a APAF-CRT study, ablation and CRT pace reduced mortality and prevented hospitalizations or deterioration due to heart failure. In earlier study, the combined approach of ablation with cardiac resynchronization therapy exhibited superiority over pharmacological therapy, resulting in reduced NYHA stadium, incidences of heart failure and hospitalization, along with improvements in the quality of life among elderly patients with permanent AF and a narrow QRS complex [2,9]. More recently, the pace and ablate strategy has demonstrated greater efficacy than pharmacological interventions in reducing mortality among patients admitted for heart failure with permanent AF, irrespective of their baseline left ventricular ejection fraction [2].

AVNA is a technically straightforward catheter ablation procedure characterized by a high rate of both acute and long-term success. Ideally, the aim of the procedure is to eliminate the compact AV node conduction, creating a stable junctional escape rhythm. Typically, this procedure involves the application of RF energy near the AV node in the right atrium, but it can also be accomplished through the left ventricle using a retrograde aortic or transseptal approach [10,11].

AVNA is a traditionally fluoroscopy-guided approach employed for the invasive treatment of patients with refractory rate-controlled permanent AF. Radiation exposure may increase the risk of dermatitis, cataracts, and cognitive decline, as well as raise the probability of cancer development [12,13,14]. Several studies have indicated that the areas most exposed in interventional cardiologists are the head, thyroid, and eye—radiation exposure is twice as high on left side than right—thus, the incidence for left-sided brain tumors and other malignancies is 2–3 times higher compared to radiologists. This translates to an accumulated risk of one additional cancer diagnosis for every 100 individuals exposed over the course of a complete professional career [6,13,14]. Moreover, a heightened occurrence of musculoskeletal pain has been reported among interventional cardiologists due to the utilization of lead aprons, which protect themselves from ionizing radiation, compared to non-interventional cardiologists [15,16]. Therefore, it is imperative to minimize radiation exposure in accordance with the ALARA principle, which aims to diminish ionizing radiation exposure to the lowest achievable level [17,18,19].

In consideration of the radiation-related risk, several technological strategies have been developed in the past decades to reduce hazardous ionizing radiation exposure among both patients and medical staff [20,21,22].

The utilization of ICE serves as a valuable tool in guiding interventions and addressing challenges associated with intricate anatomical variations, and ICE guidance is beneficial for the real-time imaging of cardiac anatomical structures during ablation procedures. The real-time feedback provided by ICE facilitates the identification of key anatomical landmarks, ensuring accurate catheter placement, and thereby enhancing the efficacy and safety of the intervention and helping to reduce or replace fluoroscopy [23,24,25]. Several previous clinical studies have found that ICE guidance in catheter ablations is associated with reduced procedure duration and fluoroscopy exposure compared to procedures without ICE guidance [7,8,26,27,28].

In a meta-analysis published in 2020, which encompassed 19 clinical trials involving 2186 patients undergoing catheter ablations for various types of cardiac arrhythmias (AF, atrial flutter, ventricular arrhythmias, and others), it was demonstrated that the utilization of ICE led to significant decreases in fluoroscopy duration (average reduction of 6.95 min), decreased fluoroscopy dose, and procedural time (average reduction of 15.2 min) compared to ablations performed without ICE [29].

During catheter ablation targeting the AV node, the main goal is to provoke a permanent third-degree AV block. The AV node, situated precisely at the apex of Koch’s triangle within the right atrium, exhibits variability in its anatomical location. Theoretically, the utilization of ICE can offer potential advantage of direct visualization of the AV node region. This capability may facilitate more precise and targeted catheter ablation strategies.

Previously, a single-center study including 91 patients showed that ICE guidance in slow-pathway ablations for the treatment of AV-node reentrant tachycardia significantly reduced the mapping and ablation time, radiation exposure, as well as the total RF time and the number of RF applications compared to the fluoroscopy-only method. Notably, 25% of the patients in the fluoroscopy-only group crossed over to the ICE-guided group after failing to achieve the procedural endpoint. Subsequently, all patients were successfully treated, with comparable numbers, durations, and cumulative energies of RF applications, as observed in the ICE-guided group. The authors concluded that the utilization of ICE facilitates slow-pathway ablations, particularly in cases involving unusual anatomical considerations [8].

These findings align with our data, indicating that fluoroscopy time, overall procedure duration, and time from first-to-last ablation can be decreased by employing ICE in patients undergoing AVNA.

Two disadvantages of the ICE-guided ablation approach warrant discussion. The utilization of ICE in catheter ablation procedures necessitate an extra venous puncture, thereby potentially elevating the chances of vascular complications. Although, it is noteworthy that previous studies and our analysis showed no notable rise in vascular complications among the ICE group. These findings indicate that the utilization of ICE can be safely implemented without increasing the occurrence of vascular events. Furthermore, recent data clearly demonstrate that the application of vascular ultrasound during femoral vein puncture significantly decreases vascular complications, even in patients undergoing catheter ablation while on uninterrupted oral anticoagulant treatment [30,31,32].

Another drawback of employing an ICE-guided ablation strategy may be the additional cost of the catheter. However, recent data have shown that the reprocessing of ICE catheters is both feasible and safe, presenting a promising path for substantial economic and environmental maintenance [33].

### Limitations

Several limitations need to be addressed. Firstly, this was a single-center retrospective trial with a limited number of patients, which restricts the generalizability of the study findings. As delineated in the Section 2, the operators had no prior experience with ICE-guided AVNA, indicating that after completing the learning curve, the results could have been even more favorable. Additionally, the use of vascular ultrasound for guiding femoral vein punctures became standard practice during the study period, introducing heterogeneity regarding femoral vein puncture methods before and after 2021. Due to the high acute and long-term success rates of AVNA and the relatively limited number of patients in the sample included the study, the results are constrained in their ability to accurately reflect success rates. Finally, although crossover to potential ICE usage was not an exclusion criterion, none occurred during the study period, preventing examination of its effect on procedural data.

## 5. Conclusions

In our retrospective single-center trial including 62 patients undergoing AVNA, we found that ICE guidance resulted in reductions in procedure duration and fluoroscopy exposure in contrast to the standard fluoroscopy-guided method, without compromising acute and long-term success rates or increasing complication rates.

## Figures and Tables

**Table 1:** Patients’ characteristics.

	ICE Group (*n* = 28)	Standard Group (*n* = 34)	*p*
Age (years)	72.3 ± 9	68.3 ± 8.8	0.08
Male (%)	18 (64.3)	20 (58.8)	0.66
Hypertension (%)	23 (82.1)	31 (91.2)	0.45
Diabetes mellitus (%)	14 (50)	18 (45)	0.36
Heart failure (%)	27 (96.5)	28 (82.4)	0.17
Coronary artery disease (%)	14 (50)	16 (47.1)	0.82
Chronic kidney disease (%)	4 (14.3)	1 (2.9)	0.17
Prior stroke/TIA (%)	1 (3.5)	2 (5.9)	0.32

Abbreviations: ICE: intracardiac echocardiography; TIA: transient ischemic attack.

**Table 2:** Summary of outcomes.

	ICE Group (*n* = 28)	Standard Group (*n* = 34)	*p*
Procedure time (min)	40 [34; 55]	60 [45; 110]	0.02
Fluoroscopy time (min)	0.30 [0.06; 0.85]	7.95 [3.23; 6.59]	<0.01
Total ablation time (min)	199 [91; 436]	294 [110; 659]	0.22
From first to the last ablation time (min)	4 [2; 16.3]	26.5 [2.3; 72.5]	0.02
Total ablation energy (J)	8272 [4004; 14,651]	6065 [2708; 16,406]	0.28
Acute success (%)	100%	94%	0.49
Complication (n)	1	0	0.94
Recurrence	1	1	1

Abbreviation: ICE: intracardiac echocardiography.

## Data Availability

The data presented in this study are available upon request from the corresponding author. The data are not publicly available due to Hungarian legal regulations.

## Supplementary Materials

The following supporting information can be downloaded at: https://www.mdpi.com/article/10.3390/jcm13154565/s1.
